# The evolution of the ventilatory ratio is a prognostic factor in mechanically ventilated COVID-19 ARDS patients

**DOI:** 10.1186/s13054-021-03727-x

**Published:** 2021-09-13

**Authors:** Antoni Torres, Anna Motos, Jordi Riera, Laia Fernández-Barat, Adrián Ceccato, Raquel Pérez-Arnal, Dario García-Gasulla, Oscar Peñuelas, José Angel Lorente, Alejandro Rodriguez, David de Gonzalo-Calvo, Raquel Almansa, Albert Gabarrús, Rosario Menéndez, Jesús F. Bermejo-Martin, Ricard Ferrer, Rosario Amaya Villar, José M. Añón, Carme Barberà, José Barberán, Aaron Blandino Ortiz, Elena Bustamante-Munguira, Jesús Caballero, Cristina Carbajales, Nieves Carbonell, Mercedes Catalán-González, Cristóbal Galbán, Víctor D. Gumucio-Sanguino, Maria del Carmen de la Torre, Emili Díaz, Ángel Estella, Elena Gallego, José Luis García Garmendia, José Garnacho-Montero, José M. Gómez, Arturo Huerta, Ruth Noemí Jorge García, Ana Loza-Vázquez, Judith Marin-Corral, Amalia Martínez de la Gándara, Ignacio Martínez Varela, Juan López Messa, Guillermo M. Albaiceta, Mariana Andrea Novo, Yhivian Peñasco, Juan Carlos Pozo-Laderas, Pilar Ricart, Inmaculada Salvador-Adell, Angel Sánchez-Miralles, Susana Sancho Chinesta, Lorenzo Socias, Jordi Solé-Violan, Fernando Suares Sipmann, Luis Tamayo Lomas, José Trenado, Ferran Barbé, CIBERESUCICOVID Project (COV20/00110, ISCIII), Berta Adell-Serrano, Alexander Agrifoglio, María Aguilar Cabello, Luciano Aguilera, Victoria Alcaraz-Serrano, Cesar Aldecoa, Cynthia Alegre, Sergio Álvarez, Antonjo Álvarez Ruiz, Rut Andrea, José Ángel, Marta Arrieta, J. Ignacio Ayestarán, Joan Ramon Badia, Mariona Badía, Orville Báez Pravia, Ana Balan Mariño, Begoña Balsera, Laura Barbena, Enric Barbeta, Tommaso Bardi, Patricia Barral Segade, Marta Barroso, José Ángel Berezo García, Judit Bigas, Rafael Blancas, María Luisa Blasco Cortés, María Boado, María Bodi Saera, Neus Bofill, María Teresa Bouza Vieiro, Leticia Bueno, Juan Bustamante-Munguira, Lucia Cachafeiro, David Campi Hermoso, Sandra Campos Fernández, Iosune Cano, Maria Luisa Cantón-Bulnes, Pablo Cardina Fernández, Laura Carrión García, Sula Carvalho, Núria Casacuberta-Barberà, Manuel Castellà, Andrea Castellví, Pedro Castro, Ramon Cicuendez Ávila, Catia Cillóniz, Luisa Clar, Cristina Climent, Jordi Codina, Pamela Conde, Sofía Contreras, María Cruz Martin, Raul de Pablo Sánchez, Diego De Mendoza, Cecilia del Busto Martínez, Yolanda Díaz, María Digna Rivas Vilas, Cristina Dólera Moreno, Irene Dot, Pedro Enríquez Giraudo, Inés Esmorís Arijón, Teresa Farre Monjo, Javier Fernández, Carlos Ferrando, Albert Figueras, Eva Forcadell-Ferreres, Lorena Forcelledo Espina, Nieves Franco, Àngels Furro, Felipe García, Beatriz García, Emilio García Prieto, Carlos García Redruello, Amaia García Sagastume, Maria Luisa Gascón Castillo, Gemma Gomà, Vanesa Gómez Casal, Silvia Gómez, Carmen Gómez Gonzalez, Jessica González, Federico Gordo, Maria Pilar Gracia, Alba Herraiz, Rubén Herrán-Monge, Mercedes Ibarz, Silvia Iglesias, Maria Teresa Janer, Gabriel Jiménez, Mar Juan Díaz, Karsa Kiarostami, Juan I. Lazo Álvarez, Miguel León, Alexandre López-Gavín, Ana López Lago, Desire Macias Guerrero, Nuria Mamolar Herrera, Rafael Mañez Mendiluce, Cecilia L. Mantellini, Gregorio Marco Naya, Pilar Marcos, Enrique Marmol Peis, Paula Martín Vicente, María Martínez, Carmen Eulalia Martínez Fernández, Maria Dolores Martínez Juan, Juan Fernando Masa Jimenez, Joan Ramon Masclans, Emilio Maseda, Eva María Menor Fernández, Mar Miralbés, Josman Monclou, Juan Carlos Montejo-González, Neus Montserrat, María Mora Aznar, Pedro Moral-Parras, Dulce Morales, Sara Guadalupe Moreno Cano, David Mosquera Rodríguez, Rosana Muñoz-Bermúdez, José María Nicolás, Ramon Nogue Bou, Rafaela Nogueras Salinas, Marta Ocón, Ana Ortega, Sergio Ossa, Pablo Pagliarani, Anna Parera Pous, Francisco Parrilla, Leire Pérez Bastida, Purificación Pérez, Gloria Pérez Planelles, Eva Pérez Rubio, David Pestaña Laguna, Àngels Piñol-Tena, Javier Prados, Andrés Pujol, Núria Ramon Coll, Gloria Renedo Sanchez-Giron, Ferran Roche-Campo, Laura Rodriguez, Felipe Rodríguez de Castro, Silvia Rodríguez, Covadonga Rodríguez Ruiz, Jorge Rubio, Alberto Rubio López, Miriam Ruiz Miralles, Pablo Ryan Murúa, Eva Saborido Paz, Ana Salazar Degracia, Miguel Sanchez, Ana Sánchez, Bitor Santacoloma, Maria Teresa Sariñena, Marta Segura Pensado, Lidia Serra, Mireia Serra-Fortuny, Ainhoa Serrano Lázaro, Lluís Servià, Laura Soliva, Carla Speziale, Daniel Tognetti, Adrián Tormos, Mateu Torres, Sandra Trefler, Javier Trujillano, Alejandro Úbeda, Luis Urrelo-Cerrón, Estela Val, Luis Valdivia Ruiz, Montserrat Vallverdú, Maria Van der Hofstadt Martin-Montalvo, Sabela Vara Adrio, Nil Vázquez, Javier Vengoechea, Pablo Vidal Cortes, Clara Vilà-Vilardel, Judit Vilanova, Tatiana Villada Warrington, Hua Yang, Minlan Yang, Ana Zapatero

**Affiliations:** 1 Centro de Investigación Biomedica En Red – Enfermedades Respiratorias (CIBERES), Barcelona, Spain; 2 Institut d’Investigacions August Pi i Sunyer (IDIBAPS), Universitat de Barcelona, Barcelona, Spain; 3 Intensive Care Department, Hospital Universitari Vall d’Hebron, Vall d’Hebron Institut de Recerca, Barcelona, Spain; 4 Barcelona Supercomputing Center (BSC), Barcelona, Spain; 5 Hospital Universitario de Getafe, Universidad Europea, Madrid, Spain; 6 Critical Care Department, Hospital Joan XXIII, Tarragona, Spain; 7 Translational Research in Respiratory Medicine, Respiratory Department, Hospital Universitari Aranu de Vilanova and Santa Maria, IRBLleida, Lleida, Spain; 8 Hospital Universitario Río Hortega de Valladolid, Valladolid, Spain; 9 Instituto de Investigación Biomédica de Salamanca (IBSAL), Gerencia Regional de Salud de Castilla y León, Salamanca, Spain; 10 Pulmonary Department, University and Polytechnic Hospital La Fe, Valencia, Spain; 11 Intensive Care Clinical Unit, Hospital Universitario Virgen de Rocío, Sevilla, Spain; 12 Servicio de Medicina Intensiva, Hospital Universitario La Paz, IdiPAZ, Madrid, Spain; 13 Hospital Santa Maria, IRBLleida, Lleida, Spain; 14 Hospital Universitario HM Montepríncipe, Universidad San Pablo-CEU, Madrid, Spain; 15 Servicio de Medicina Intensiva, Hospital Universitario Ramón y Cajal, Madrid, Spain; 16 Department of Intensive Care Medicine, Hospital Clínico Universitario Valladolid, Valladolid, Spain; 17 Critical Care Department, Hospital Universitari Arnau de Vilanova, IRBLleida, Lleida, Spain; 18 Hospital Álvaro Cunqueiro, Vigo, Spain; 19 Intensive Care Unit, Hospital Clínico y Universitario de Valencia, Valencia, Spain; 20 Department of Intensive Care Medicine, Hospital Universitario, 12 de Octubre, Madrid, Spain; 21 Department of Medicine, CHUS, Complejo Hospitalario Universitario de Santiago, Santiago de Compostela, Spain; 22 Department of Intensive Care, Hospital Universitari de Bellvitge, L’Hospitalet de Llobregat, Barcelona, Spain; 23 Bellvitge Biomedical Research Institute (IDIBELL), L’Hospitalet de Llobregat, Barcelona, Spain; 24 Hospital de Mataró de Barcelona, Barcelona, Spain; 25 Department of Medicine, Universitat Autònoma de Barcelona (UAB), Barcelona, Spain; 26 Critical Care Department, Corporació Sanitària Parc Taulí, Sabadell, Barcelona, Spain; 27 Departamento Medicina Facultad Medicina, Universidad de Cádiz, Hospital Universitario de Jerez, Jerez de la Frontera, Spain; 28 Unidad de Cuidados Intensivos, Hospital San Pedro de Alcántara, Cáceres, Spain; 29 Intensive Care Unit, Hospital San Juan de Dios del Aljarafe, Sevilla, Spain; 30 Intensive Care Clinical Unit, Hospital Universitario Virgen Macarena, Seville, Spain; 31 Hospital General Universitario Gregorio Marañón, Madrid, Spain; 32 Pulmonary and Critical Care Division, Emergency Department, Clínica Sagrada Família, Barcelona, Spain; 33 Intensive Care Department, Hospital Nuestra Señora de Gracia, Zaragoza, Spain; 34 Unidad de Medicina Intensiva, Hospital Universitario Virgen de Valme, Sevilla, Spain; 35 Critical Care Department, Hospital del Mar-IMIM, Barcelona, Spain; 36 Department of Intensive Medicine, Hospital Universitario Infanta Leonor, Madrid, Spain; 37 Critical Care Department, Hospital Universitario Lucus Augusti, Lugo, Spain; 38 Complejo Asistencial Universitario de Palencia, Palencia, Spain; 39 Departamento de Biología Funcional, Instituto Universitario de Oncología del Principado de Asturias, Universidad de Oviedo, Oviedo, Spain; 40 Instituto de Investigación Sanitaria del Principado de Asturias, Hospital Central de Asturias, Oviedo, Spain; 41 Servei de Medicina Intensiva, Hospital Universitari Son Espases, Palma de Mallorca, Illes Balears, Spain; 42 Servicio de Medicina Intensiva, Hospital Universitario Marqués de Valdecilla, Santander, Spain; 43 UGC-Medicina Intensiva, Hospital Universitario Reina Sofia, Instituto Maimonides IMIBIC, Córdoba, Spain; 44 Servei de medicina intensiva, Hospital Universitari Germans Trias, Badalona, Spain; 45 Hospital Verge de La Cinta, Tortosa, Tarragona, Spain; 46 Hospital de Sant Joan d’Alacant, Alacant, Spain; 47 Servicio de medicina intensiva, Hospital Universitario y Politécnico La Fe, Valencia, Spain; 48 Intensive Care Unit, Hospital Son Llàtzer, Palma de Mallorca, Illes Balears, Spain; 49 Critical Care Department, Hospital Dr. Negrín Gran Canaria, Las Palmas, Gran Canaria, Spain; 50 Intensive Care Unit, Hospital Universitario La Princesa, Madrid, Spain; 51 Critical Care Department, Hospital Universitario Río Hortega de Valladolid, Valladolid, Spain; 52 Servicio de Medicina Intensiva, Hospital Universitario Mútua de Terrassa, Terrassa, Barcelona, Spain; 53 Servei de Pneumologia i Al·lèrgia Respiratòria, Hospital Clínic, Villarroel 170, Esc 6/8 Planta 2, 08036 Barcelona, Spain

**Keywords:** Ventilatory ratio, Mechanical ventilation, COVID-19, SARS-CoV-2, Coronavirus

## Abstract

**Background:**

Mortality due to COVID-19 is high, especially in patients requiring mechanical ventilation. The purpose of the study is to investigate associations between mortality and variables measured during the first three days of mechanical ventilation in patients with COVID-19 intubated at ICU admission.

**Methods:**

Multicenter, observational, cohort study includes consecutive patients with COVID-19 admitted to 44 Spanish ICUs between February 25 and July 31, 2020, who required intubation at ICU admission and mechanical ventilation for more than three days. We collected demographic and clinical data prior to admission; information about clinical evolution at days 1 and 3 of mechanical ventilation; and outcomes.

**Results:**

Of the 2,095 patients with COVID-19 admitted to the ICU, 1,118 (53.3%) were intubated at day 1 and remained under mechanical ventilation at day three. From days 1 to 3, PaO_2_/FiO_2_ increased from 115.6 [80.0–171.2] to 180.0 [135.4–227.9] mmHg and the ventilatory ratio from 1.73 [1.33–2.25] to 1.96 [1.61–2.40]. In-hospital mortality was 38.7%. A higher increase between ICU admission and day 3 in the ventilatory ratio (OR 1.04 [CI 1.01–1.07], *p* = 0.030) and creatinine levels (OR 1.05 [CI 1.01–1.09], *p* = 0.005) and a lower increase in platelet counts (OR 0.96 [CI 0.93–1.00], *p* = 0.037) were independently associated with a higher risk of death. No association between mortality and the PaO_2_/FiO_2_ variation was observed (OR 0.99 [CI 0.95 to 1.02], *p* = 0.47).

**Conclusions:**

Higher ventilatory ratio and its increase at day 3 is associated with mortality in patients with COVID-19 receiving mechanical ventilation at ICU admission. No association was found in the PaO_2_/FiO_2_ variation.

**Supplementary Information:**

The online version contains supplementary material available at 10.1186/s13054-021-03727-x.

## Background

The mortality recorded during the COVID-19 pandemic, although variable, is extremely high, especially in patients admitted to the ICU, and even more in those patients that require invasive mechanical ventilation (MV) for ARDS [1]. In a recent meta-analysis including 69 studies, the overall case fatality rate was 45% [2]. When cases were stratified by age, mortality rise to 84.4% in patients older than 80 years. However, the observed heterogeneity was high (*I* > 90%) but with a non-significant Egger regression test that suggested no publication bias. The 15 studies included from Europe gave similar results compared to other continents [2].

The Eurosurveillance registry in Spain [3] reported epidemiological data from the first wave of the pandemic in Spain up to April 17, 2020. They reported 8,289 patients admitted to ICUs (4.6% of the total), 4,085 of whom required MV (78% of those admitted to an ICU). The overall mortality of mechanically ventilated patients was 42%. Among the various factors associated with death, age, chronic kidney disease, and a shorter time between symptom onset and ED visit were the most important. The REVA network study [4] recently described 4,643 patients admitted to ICUs in France. On day 1 of admission, 63% of patients were intubated and overall, 80% received MV. The 90-day mortality was associated with age, diabetes, obesity, and severe ARDS.

The majority of investigations dealing with COVID patients admitted to the ICU have attempted to determine prognostic factors using data from the first day of hospital admission or data obtained on day 1 of ICU admission. In ICU patients, these data can be misinterpreted, either because the patients are not “clinically stable” or because they are not relevant as they cannot be modified (e.g., age, obesity, etc.). On the other hand, in many respiratory acute diseases with acute respiratory failure, the patient’s status 72 h after ICU admission gives important clues about prognosis, taking into account what has happened since admission, including adequate or inadequate management [5, 6]. This type of observational research can provide evidence about beneficial medical interventions.

CIBERESUCICOVID (Centro de Investigación Biomédica En Red de Enfermedades ReSpiratoria—Factores de riesgo y pronóstico personalizados y seguimiento a un año de los enfermos ingresados en las Unidades de Cuidados Intensivos españolas infectados por COVID-19) is a multicenter observational study (NCT04457505) that is studying patients admitted to an ICU due to SARS-CoV-2 infection [7]. Patients who required invasive mechanical ventilation during the first day of admission to the ICU and who remained ventilated 3 days later were selected to capture what has happened since admission to 72 h after ICU admission. Our hypothesis was that the differences between day 1 and day 3 in relevant and independently associated variables with in-hospital mortality may help clinicians both to establish corrective measures and to have a better understanding of the prognosis.

## Methods

### Study design

CIBERESUCICOVID is a multicenter, observational, prospective/retrospective cohort study that enrolled patients with COVID-19 infection admitted to the Spanish ICUs (participating centers are listed in the Additional file 1: Table 1). The study was approved by the Institution’s Internal Review Board (Comité Ètic d’Investigació Clínica, registry number HCB/2020/0370), and informed consent was obtained from either patients or their relatives. Local researchers were contacted by a member of the study team and participating hospitals obtained local ethics committee approval. Data collection was started in May 2020. Consecutive patients admitted before the start of the study were included retrospectively. Patients admitted after the start of the study were included prospectively. De-identified patient data were collected and stored via the REDCap electronic data capture tool, hosted at the Centro de Investigación Biomédica en Red (CIBER), Spain. Data from patients’ medical records were incorporated into a separate database by trained local researchers. Prior to statistical analyses, the data were checked by three independent experienced data collectors trained in critical care (PC, AM, CS), and site investigators were contacted with any queries. Missing analyses were performed, and site investigators were contacted in order to obtain reliable and complete data as much as possible (Additional file 1: Fig. 1). Results were reported in accordance with the Strengthening the Reporting of Observational Studies in Epidemiology (STROBE) guidelines [8].

**Table 1:** Demographic and clinical characteristics of patients that received invasive mechanical ventilation (MV) during the first 24 h of ICU admission

	No	All patients (*n* = 1118)	Survivors (*n* = 685)	Non-survivors (*n* = 433)	*p*-value
Age, years	1118	65.0 [57.0–72.0]	62.0 [53.0–69.0]	69.0 [63.0–74.0]	** < 0.001**
Age, categories	1118				
< 50		144 (12.9%)	125 (18.3%)	19 (4.4%)	** < 0.001**
50–69		612 (54.7%)	407 (59.4%)	205 (47.3%)	** < 0.001**
70–79		345 (31.0%)	149 (21.8%)	197 (45.5%)	** < 0.001**
≥ 80		16 (1.4%)	4 (0.6%)	12 (2.8%)	**0.004**
Sex, female	1118	322 (28.8%)	211 (30.8%)	111 (25.6%)	0.07
BMI, kg/m^2^	1019	28.1 [25.6–31.5]	28.3 [25.7–31.6]	27.8 [25.5–31.3]	0.27
Comorbidities					
Active smoker	741	61 (8.2%)	33 (7.1%)	28 (10.4%)	0.17
Hypertension	1117	555 (49.7%)	308 (45.0%)	247 (57.2%)	** < 0.001**
Diabetes mellitus	1117	258 (23.1%)	153 (22.3%)	105 (24.3%)	0.47
Dyslipidemia	1117	275 (24.6%)	165 (24.1%)	110 (25.4%)	0.62
Chronic cardiac failure	1117	138 (12.4%)	60 (8.8%)	78 (18.1%)	** < 0.001**
Chronic kidney disease	1117	58 (5.1%)	25 (3.6%)	33 (7.4%)	**0.003**
Chronic respiratory disease	1117	119 (10.7%)	52 (7.6%)	67 (15.5%)	** < 0.001**
Days since first symptoms	1098	7.0 [5.0–9.0]	7.0 [5.0–9.0]	7.0 [4.0–9.0]	**0.047**
Days from hospital admission to intubation	1116	1.0 [0.0–4.0]	1.0 [0.0–3.0]	1.0 [0.0–4.0]	0.38
APACHE score	615	12.0 [9.5–16.0]	11.0 [8.8–15.0]	14.0 [11.0–18.0]	** < 0.001**
SOFA score	779	7.0 [5.0–8.0]	7.0 [4.0–8.0]	7.0 [5.0–9.0]	** < 0.001**
SOFA hemodynamic component	1041	3.0 [0.0–4.0]	3.0 [0.0–4.0]	3.0 [0.0–4.0]	0.09
SOFA renal component	1108	0.0 [0.0–0.0]	0.0 [0.0–0.0]	0.0 [0.0–1.0]	** < 0.001**
Temperature, ºC	995	36.9 [36.0–37.8]	37.0 [36.0–37.9]	36.8 [36.0–37.8]	0.08
Respiratory rate, bpm	942	25.0 [20.0–30.0]	24.0 [20.0–30.0]	25.0 [20.0–30.0]	0.63
Arterial blood gases at ICU admission					
PaO_2_/FiO_2_ ratio, mmHg	1067	115.6 [80.0–171.2]	117.5 [82.2–176.7]	111.27 [74.1–157.9]	**0.017**
PaO_2_/F_I_O_2_ ratio categories	1067				
PaO_2_/FiO_2_ ratio < 100 mmHg		424 (39.7%)	249 (37.4%)	175 (43.5%)	**0.05**
PaO_2_/FiO_2_ ratio ≥ 100 and < 200 mmHg		462 (43.3%)	295 (44.4%)	167 (41.5%)	0.37
PaO_2_/FiO_2_ ratio ≥ 200 and < 300 mmHg		132 (12.4%)	88 (13.2%)	44 (11.0%)	0.29
PaO_2_/FiO_2_ ratio ≥ 300 mmHg		49 (4.6%)	33 (5.0%)	16 (4.0%)	0.55
pH	1076	7.36 [7.29–7.43]	7.38 [7.31–7.44]	7.33 [7.26–7.41]	** < 0.001**
PaCO_2_, mmHg	1084	43.9 [36.0–52.0]	43.0 [36.0–50.5]	45.1 [37.0–55.0]	**0.001**
Lactate, mg/dL	753	13.0 [9.9–17.1]	12.6 [9.0–16.2]	14.4 [10.8–18.9]	** < 0.001**
Laboratory findings at ICU admission					
Lymphocyte count, 10^9^/L	1095	0.63 [0.43–0.90]	0.67 [0.47–0.95]	0.60 [0.40–0.84]	**0.001**
Neutrophil count, 10^9^/L	472	7.66 [5.57–11.32]	7.32 [5.30–10.84]	7.97 [5.81–12.00]	0.10
Platelet count, 10^9^/L	1106	226.0 [173.0–299.0]	236.0 [182.0–308.8]	212.0 [163.8–277.3]	** < 0.001**
D-dimers, mg/L	877	1.12 [0.59–3.52]	0.98 [0.53–2.30]	1.71 [0.70–5.51]	** < 0.001**
Ferritin, ng/mL	379	1398 [821–2250]	1330 [766–2172]	1500 [890–2400]	0.11
IL6, pg/mL	215	114.0 [47.5–181.4]	103.1 [45.1–186.8]	125.9 [60.2–153.2]	0.66
CRP, mg/dL	987	17.5 [9.4–26.6]	17.0 [9.1–26.0]	18.4 [10.0–27.7]	0.068
Bilirubin, mg/dL	1018	0.62 [0.42–1.00]	0.64 [0.43–1.00]	0.60 [0.40–1.00]	0.76
Serum creatinine, mg/dL	1108	0.87 [0.67–1.15]	0.82 [0.64–1.07]	0.95 [0.75–1.28]	** < 0.001**
Ventilatory setting and pulmonary mechanics at MV start					
Tidal volume/PBW (mL/kg)	908	7.1 [6.4–8.0]	7.1 [6.4–7.9]	7.1 [6.4–8.0]	0.80
Respiratory rate, bpm	999	20.0 [18.0–24.0]	20.0 [18.0–24.0]	20.0 [18.0–24.0]	0.63
PEEP, cmH_2_O	1045	12.0 [10.0–14.0]	12.0 [10.0–14.0]	12.0 [10.0–14.0]	0.52
FiO_2_, %	1064	75.0 [60.0–100.0]	70.0 [60.0–100.0]	80.0 [60.0–100.0]	** < 0.001**
Peak inspiratory pressure, cmH_2_O	509	31.0 [27.0–35.0]	30.0 [27.0–34.0]	31.0 [27.5–35.5]	0.08
End-inspiratory plateau pressure, cmH_2_O	440	25.0 [22.0–28.0]	25.0 [21.0–28.0]	25.0 [22.0–28.0]	0.10
Driving pressure, cmH_2_O^a^	432	12.0 [9.6–15.0]	12.0 [9.0–14.0]	12.0 [9.8–15.0]	0.25
Compliance, mL/cmH_2_O^b^	413	37.1 [29.3–50.0]	37.1 [30.0–50.0]	35.8 [28.2–50.0]	0.33
Ventilatory ratio^c^	889	1.73 [1.33–2.25]	1.68 [1.32–2.15]	1.87 [1.41–2.36]	**0.001**
Position	960				
Supine		591 (61.6%)	394 (65.5%)	197 (55.0%)	** < 0.001**
Prone		346 (36.0%)	192 (31.9%)	154 (43.0%)	**0.008**
Other		23 (2.4%)	16 (2.6%)	7 (2.0%)	0.46

Continuous variables are expressed as median (IQR) and categorical variables as number (percentages). P-values marked in bold indicate numbers that are significant on the 95% confidence limit. *CRP* C-reactive protein; *FiO*_*2*_ fraction of inspired oxygen; *MV* mechanical ventilation; *PaCO*_*2*_ arterial partial pressure of carbon dioxide; *PaO*_*2*_ partial pressure of arterial oxygen; *PBW* predicted body weight; *SOFA* sequential organ failure assessment score

^a^Defined as plateau pressure—PEEP

^b^Defined as tidal volume/(Plateau pressure − PEEP)

^c^Defined as (minute ventilation × PaCO_2_)/(PBW × 100 × 37.5)

**Fig. 1:**
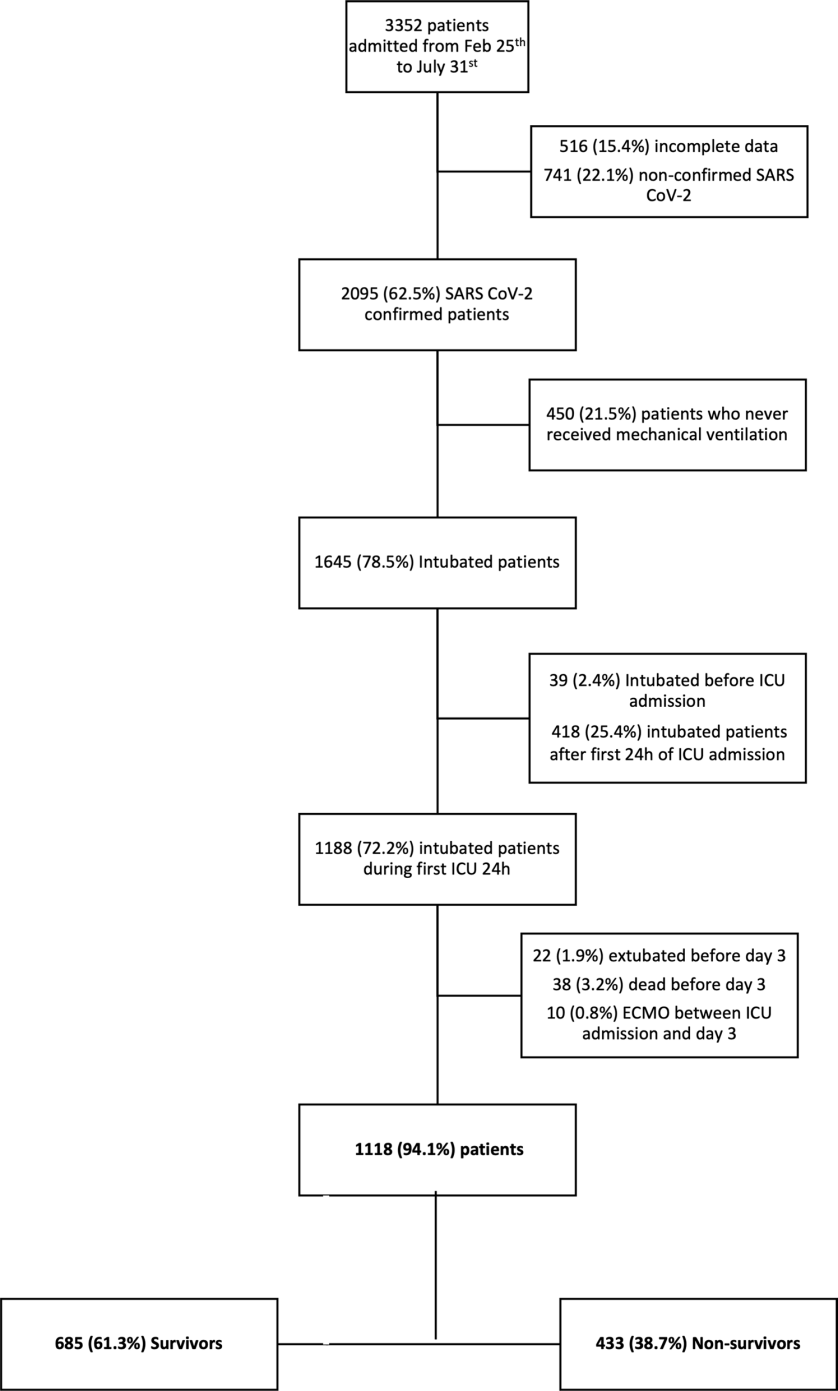
Flowchart of patient screening and enrollment. A total of 1118 patients were followed-up until hospital discharge or death. *ECMO* extracorporeal membrane oxygenation; *ICU* intensive care unit; *SARS-CoV-2* severe acute respiratory syndrome coronavirus 2

### Study population and data collection

All consecutive patients admitted to the ICU at a participating center from February 25 to July 31, 2020, were enrolled if they fulfilled the following criteria: ≥ 18 years old, admission to ICU, and laboratory confirmed SARS-CoV-2 infection. For this study, we selected those patients who required invasive mechanical ventilation during the first day of admission to the ICU and who remained ventilated 3 days later. This selection responded to capture what has happened during first days of ICU admission in a homogenous population. Patients were excluded if they had non-confirmed SARS-CoV-2 infection, no data at baseline or at hospital discharge, or who were admitted to an ICU for other reasons. Patients who required ECMO support within this period (i.e., ICU admission—Day 3) were excluded from the analysis to avoid potential bias in blood gases analysis and pulmonary mechanics [9].

After enrollment, prior epidemiological data including demographics, comorbidities, clinical symptoms, disease chronology, and treatment administered upon hospital admission were collected. The site researchers subsequently collected data acquired at hospital admission, ICU admission, start of MV, 72–96 h after ICU admission, weaning, ICU discharge and hospital discharge, including vital signs, respiratory support devices (i.e., oxygen mask, high flow nasal cannular, and noninvasive and invasive mechanical ventilation), the use of adjunctive therapies (i.e., neuromuscular blockade, prone position, and recruitment maneuvers), laboratory findings, arterial blood gases, and mechanical ventilation settings if appropriate. Hemodynamic parameters and organ dysfunction were studied with the Sequential Organ Assessment Failure Score (SOFA) at ICU admission. The pharmacological treatments administered upon and during ICU admission until either discharge from the ICU or hospital, or death, were also collected.

Specific data regarding MV since the start of intubation, as well as, at day 3 were analyzed. MV parameters related to ventilation-induced lung injury (VILI) such as tidal volume, respiratory rate, end-inspiratory plateau and peak inspiratory pressures, positive end-expiratory pressure (PEEP), driving pressure, and static compliance of the respiratory system (Crs) were collected. Impairment in oxygenation was analyzed using the PaO_2_/FiO_2_ ratio and abnormalities of CO_2_ metabolism were studied using the ventilatory ratio (VR), a surrogate parameter of Vd/Vt. The worst event values were preferentially recorded.

### Definitions

The diagnosis of ARDS was based on the Berlin definition [10]. Chronic respiratory disease was defined as any chronic obstructive pulmonary disease, cystic fibrosis, bronchiectasis, and interstitial lung diseases, excluding asthma [11]. Tidal volume was reported in mL/kg of predicted body weight (PBW). Driving pressure was defined as plateau pressure minus PEEP. Crs was calculated as tidal volume/ (plateau pressure − PEEP). Ventilatory ratio was defined as (minute ventilation x PaCO_2_)/(PBW × 100 × 37.5) (normal value: 1). The delta measurements were computed as the difference between the value at day 3 of ICU admission and day 1 of ICU admission. Other definitions were reported in the Online Supplementary Data.

### Outcomes

The primary outcome was in-hospital mortality. ICU mortality, 28 days mortality, the duration of ventilation, ICU, and hospital length of stay were also collected and reported. The pulmonary (i.e., nosocomial pneumonia, tracheobronchitis, ARDS, pneumothorax, pleural effusion, pulmonary embolism) and extrapulmonary complications (including complications that affect hematologic, cardiovascular, renal, gastrointestinal, hepatic, endocrinologic, and neurologic systems) during ICU admission were also collected and included at hospital discharge.

### Statistical analysis

We report the number and percentage of patients for categorical variables and the median [first quartile − third quartile] for continuous variables. Percentages were calculated excluding missing data. Categorical variables were compared using the Chi-squared test or Fisher’s exact test, whereas continuous variables were compared using the nonparametric Mann–Whitney U test.

To explore the risk factors associated with in-hospital death, mixed-effects multivariable models [12, 13] were used, defined by a binomial probability distribution and a logit link function, with centers as a random effect. The following variables were included in the first multivariable model based on clinical relevance only: age, sex, hypertension, chronic respiratory disease, SOFA hemodynamic component, and at ICU admission: PaO_2_/FiO_2_ ratio, serum creatinine, lymphocyte count, platelet count, D-dimer, total bilirubin, and ventilatory ratio. The following variables were included in the second multivariable model based on previous findings and clinical constraints: age, delta PaO_2_/FiO_2_ ratio, delta serum creatinine, delta lymphocyte count, delta platelet count, delta total bilirubin, and delta ventilatory ratio. Both models included the relevant ventilatory and oxygenation variables, laboratory tests, organ support, and demographic characteristics. Odds ratios (ORs) and their 95% confidence intervals were calculated. Single collinearity was evaluated using the Pearson’s coefficient correlation (*r*). SOFA was excluded because of collinearity (*r* >|± 0.30|) with creatinine, while PaCO_2_ and pH were omitted because ventilatory ratio collinearity. Multicollinearity was examined by means of the variance inflation factor (VIF). A rule of thumb is that if VIF > 10, then multicollinearity is high [14]. The linearity of continuous variables was assessed using the Box–Tidwell test (Additional file 1: Table 2). The linearity assumption for delta measurements was confirmed in a scatter plot of the predictor against the logit because the Box–Tidwell test does not accept negative values (Additional file 1: Fig. 2). Variables not satisfying this criterion were entered as restricted cubic splines in the model. The receiver operating characteristic curve was used to assess the discriminatory ability of the model to distinguish dead patients from living patients and is expressed as the area under the receiver operating characteristic curve (AUC), ranging from 0.5 (no discriminative ability) to 1.0 (perfect discriminative ability). Calibration was assessed using the Brier score, ranging from 0.0 to 1.0, where a model with perfect skill has a score of 0.0 and the worst has a score of 1.0. The level of significance was set at 0.05 (two-tailed), and all analyses were performed using R version 4.0.3.

**Table 2:** Laboratory findings and ventilation management of the early ventilated patients at day 3 according to in-hospital mortality

	No	All patients (*n* = 1118)	Survivors (*n* = 685)	Non-survivors (*n* = 433)	*p*-value
Arterial blood gases					
PaO_**2**_/FiO_**2**_ ratio, mmHg	1054	180.0 [135.4–227.9]	190.0 [148.6–237.5]	158.0 [114.0–208.7]	** < 0.001**
PaO_2_/FiO_2_ ratio categories	1054				
PaO_**2**_/FiO_**2**_ ratio < 100 mmHg		98 (9.3%)	36 (5.6%)	63 (15.2%)	** < 0.001**
PaO_**2**_/FiO_**2**_ ratio ≥ 100 and < 200 mmHg		550 (52.2%)	317 (49.4%)	233 (56.6%)	**0.023**
PaO_**2**_/FiO_**2**_ ratio ≥ 200 and < 300 mmHg		313 (29.7%)	232 (36.1%)	81(19.7%)	** < 0.001**
PaO_**2**_/FiO_**2**_ ratio ≥ 300 mmHg		91 (8.6%)	56 (8.7%)	35 (8.5%)	1.00
pH	1082	7.39 [7.33–7.44]	7.40 [7.35–7.45]	7.37 [7.30–7.41]	** < 0.001**
PaCO_**2**_, mmHg	1090	47.0 [41.8–54.0]	46.0 [40.0–51.0]	50.3 [44.0–58.0]	** < 0.001**
Lactate, mg/dL	756	15.3 [11.7–20.1]	15.3 [11.1–19.8]	16.6 [12.6–20.7]	**0.001**
Laboratory findings					
Lymphocyte count, 10^9^/L	1090	0.64 [0.41–1.00]	0.70 [0.48–1.00]	0.60 [0.38–0.90]	** < 0.001**
Neutrophil count, 10^9^/L	472	7.58 [5.60–10.50]	7.40 [5.24–10.29]	8.10 [6.10–10.5]	**0.030**
Platelet count, 10^9^/L	1095	255.0 [189.0–325.0]	274.0 [206.5–340.0]	222.5 [168.8–293.0]	** < 0.001**
D-dimers, mg/L	754	2.31 [1.05–6.52]	1.97 [0.96–4.76]	3.27 [1.39–8.92]	** < 0.001**
Ferritin, ng/mL	345	1326 [835–2200]	1308 [790–2144]	1361 [839–2244]	0.50
IL6, pg/mL	115	98.0 [43.1–196.6]	81.0 [44.4–191.0]	125.9 [41.7–196.9]	0.51
CRP, mg/dL	925	10.0 [3.4–22.6]	9.3 [3.1–20.7]	10.8 [3.8–23.5]	0.09
Bilirubin, mg/dL	957	0.75 [0.42–1.30]	0.75 [0.42–1.26]	0.76 [0.42–1.35]	0.71
Serum creatinine, mg/dL	1101	0.92 [0.67–1.39]	0.81 [0.62–1.17]	1.10 [0.78–1.88]	** < 0.001**
Ventilatory setting and pulmonary mechanics					
Tidal volume/PBW (ml/kg)	926	7.3 [6.5–8.1]	7.3 [6.5–8.1]	7.3 [6.5–8.1]	0.81
Respiratory rate, rpm	1006	22.0 [18.0–24.0]	21.0 [18.0–24.0]	22.0 [20.0–25.0]	**0.003**
PEEP, cmH_2_O	1051	12.0 [10.0–14.0]	12.0 [10.0–14.0]	12.0 [10.0–14.0]	0.46
FiO_2_, %	1065	50.0 [40.0–60.0]	50.0 [40.0–60.0]	60.0 [50.0–70.0]	** < 0.001**
Peak inspiratory pressure, cmH_2_O	537	30.0 [27.0–35.0]	30.0 [27.8–34.6]	33.0 [27.0–36.0]	0.11
End-inspiratory plateau pressure, cmH_2_O	413	24.0 [21.0–28.0]	24.0 [21.0–27.0]	25.0 [22.0–29.0]	**0.001**
Driving pressure, cmH_2_O^a^	401	12.0 [10.0–15.0]	12.0 [9.5–14.0]	12.2 [10.0–16.0]	**0.022**
Compliance, mL/cmH_2_O^b^	399	37.5 [28.8–48.0]	38.4 [30.0–48.3]	34.6 [25.1–46.6]	**0.032**
Ventilatory ratio^c^	905	1.96 [1.61–2.40]	1.86 [1.53–2.24]	2.11 [1.77–2.68]	** < 0.001**
Position	967				
Supine		658 (68.0%)	451 (74.0%)	219 (58.6%)	** < 0.001**
Prone		285 (29.5%)	138 (23.3%)	145 (38.8%)	** < 0.001**
Other		24(2.5%)	16 (2.7%)	9 (2.4%)	0.84

Continuous variables are expressed as median (IQR) and categorical variables as number (percentages). P-values marked in bold indicate numbers that are significant on the 95% confidence limit. *CRP* C-reactive protein; *FiO*_*2*_ fraction of inspired oxygen; *PaCO*_*2*_ arterial partial pressure of carbon dioxide; *PaO*_*2*_ partial pressure of arterial oxygen; *PBW* predicted body weight

^a^Defined as plateau pressure—PEEP

^b^Defined as tidal volume/(Plateau pressure − PEEP)

^c^Defined as (minute ventilation × PaCO_2_) / (PBW × 100 × 37.5)

**Fig. 2:**
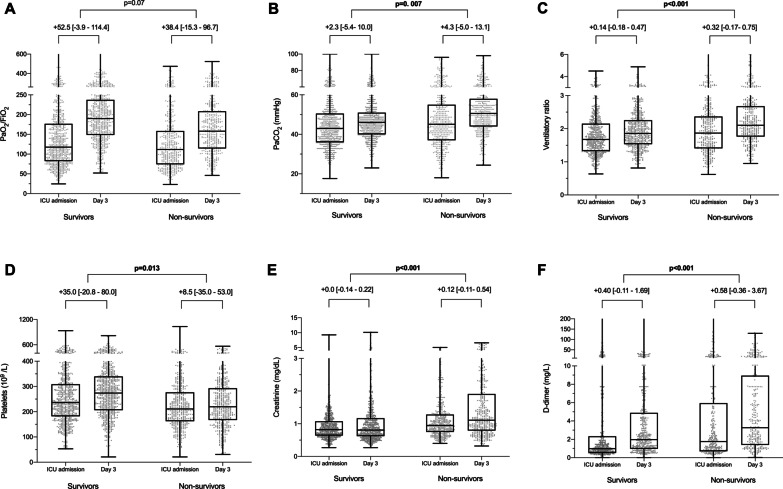
Comparison between ICU admission and day 3 stratified by survival status. Horizontal lines of the boxplots show median values, while the upper and lower lines depict the interquartile range. Dots depict values for each patient. Delta partial pressure of oxygen in arterial blood (PaO_2_) did not vary between survivor and non-survivor patients (**A**), while partial pressure of carbon dioxide in arterial blood (PaCO_2_) (**B**), the ventilatory ratio (**C**), platelets count (**D**), serum creatinine (**E**), and D-dimer (**F**) significantly differed

## Results

### Demographics and clinical characteristics at ICU admission

Between February 25 and July 31, 2020, 2,095 patients with COVID-19 were admitted to 44 ICUs (Additional file 1: Table 3), 1,645 (78.5%) required MV (Additional file 1: Table 4), and 1,188 (72.2%) needed intubation on the first day of ICU admission (Fig. 1). The majority of this last cohort still received MV support at day three of ICU admission [1,118 (94.1%)], while 38 (3.1%) died and 22 (1.8%) could be extubated during these first three days. Ten patients (0.8%) who required ECMO support during these first three days were excluded.

**Table 3:** Multivariable model assessing predictors of in-hospital mortality (*N* = 619 patients)

Variable	Odds ratio (95% CI)	*p* value
Age, years	1.01 (1.01 to 1.02)	** < 0.001**
Sex, male	1.04 (0.96 to 1.12)	0.34
Hypertension	1.01 (0.94 to 1.09)	0.83
Chronic respiratory disease	1.16 (1.03 to 1.29)	**0.013**
SOFA hemodynamic component	1.01 (0.99 to 1.03)	0.53
PaO_2_/F_i_O_2_ ratio at ICU admission, mmHg	1.00 (0.99 to 1.00)	0.14
Serum creatinine at ICU admission, mg/dL	1.09 (1.03 to 1.15)	**0.003**
Lymphocyte count at ICU admission, × 10^9^/L	1.00 (0.98 to 1.03)	0.89
Platelet count at ICU admission, × 10^9^/L	1.00 (1.00 to 1.00)	0.12
Total bilirubin at ICU admission, mg/dL	0.97 (0.91 to 1.03)	0.27
D-dimers at ICU admission, μg/L	1.00 (1.00 to 1.00)	0.29
Ventilatory ratio at ICU admission	1.07 (1.01 to 1.14)	**0.016**

Mixed-effects model with centers as a random effect and considering a binomial distribution. AUC statistic (area under the curve) is 0.79 (95% CI 0.76 to 0.83), and Brier score is 0.18. P-values marked in bold indicate numbers that are significant on the 95% confidence limit. *CI* confidence interval; *FiO*_*2*_, fraction of inspired oxygen; *ICU*, intensive care unit; *PaO*_*2*_, partial pressure of arterial oxygen; *SOFA*, sequential organ failure assessment score

**Table 4:** Multivariable model assessing predictors of in-hospital mortality (*N* = 660)

Variable	Odds ratio (95% CI)	*p* value
Age	1.01 (1.01 to 1.02)	** < 0.001**
Delta PaO_2_/F_i_O_2_ ratio, mmHg	1.00 (1.00 to 1.00)	0.47
Delta serum creatinine, mg/dL	1.06 (1.02 to 1.11)	**0.005**
Delta lymphocyte count, × 10^9^/L	1.01 (0.98 to 1.04)	0.40
Delta platelet count, × 10^9^/L	0.99 (0.98 to 1.00)	**0.037**
Delta total bilirubin, mg/dL	0.99 (0.97 to 1.02)	0.79
Delta ventilatory ratio	1.05 (1.01 to 1.10)	**0.030**

Mixed-effects model with centers as a random effect and considering a binomial distribution. AUC statistic (area under the curve) is 0.79 (95% CI 0.76 to 0.83), and Brier score is 0.18. P-values marked in bold indicate numbers that are significant on the 95% confidence limit. *CI* confidence interval; *FiO*_*2*_ fraction of inspired oxygen; *PaO*_*2*_ partial pressure of arterial oxygen

The characteristics of the study population of patients needing intubation on the first day and still needing MV of the third day are detailed in Table 1. In summary, the mean age was 65.0 [57.0–72.0] years, with the majority (54.7%) being aged between 50 and 69 years. Seventy-one percent (*n* = 786) were male, the most frequent comorbidity was hypertension, which was present in 555 (49.7%), and only 61 (8.2%) were active smokers. On the first day of MV, patients showed a respiratory system compliance of 37.1 [29.3–50.0] mL/cmH_2_O, with a driving pressure of 12.0 [9.6–15.0] cmH_2_O, a tidal volume of 7.1 [6.4–8.0] mL/Kg and a ventilatory ratio of 1.7 [1.4–2.3]. The PaO_2_/FiO_2_ ratio was 115.6 [80.0–171.2] mmHg, and the ratio was under 200 mmHg in 886 (83.0%) patients and under 100 mmHg in 424 (39.7%); 346 (36.0%) patients needed prone positioning during this first MV day. The measured respiratory rate was 20.0 [18.0–24.0] bpm, with a PaCO_2_ of 43.9 [36.0–52.0] mmHg and a pH of 7.36 [7.29–7.43]. The level of lactate was 13.0 [9.9–17.1] mg/dL with 704 (67.6%) patients needing vasoactive drugs at this time point. The SOFA score was 7.0 [5.0–8.0], the median level of creatinine was 0.87 [0.67–1.15] mg/dL, and the platelet count was 226.0 [173.0–299.0] × 10^9^/L. Regarding the COVID-19 laboratory biomarkers, the mean lymphocyte count was low (0.63 [0.43–0.90] × 10^9^/L) and the plasma levels of D-dimer, ferritin, and IL-6 were notably elevated with median values of 1.12 [0.59–3.52] mg/L, 1389.0 [821.0–2250.0] ng/mL and 114.0 [47.5–181.4] pg/mL, respectively.

### Clinical evolution at day 3 of ICU admission

The main findings at the third MV day are displayed in Table 2. Delta differences between day 3 and ICU admission showed a general increase in the PaO_2_/FiO_2_ ratio (+ 46.7 [–9.3 to 108.3]), but this increment was not significantly higher in patients that survived to hospital discharge (*p* = 0.07). On the contrary, we found differences in the changes in the level of PaCO_2_ (+ 2.3 [–5.4 to + 10.0] vs. + 4.3 [–5.0 to + 13.1] mmHg, *p* = 0.007) and the magnitude of the ventilatory ratio (+ 0.14 [–0.18 to + 0.47] vs. + 0.32 [–0.17 to + 0.75], *p* < 0.001) between survivors and non-survivors. In this regard, the delta of D-dimer was the only COVID-19 biomarker that differed significantly between survivors and non-survivors (+ 0.40 [–0.11 to + 1.69] vs. + 0.58 [–0.36 to + 3.67] mg/L, *p* = 0.001). Regarding other extrapulmonary biomarkers of organ dysfunction, the delta of creatinine levels was higher in non-survivors (+ 0.0 [–0.14 to + 0.22] vs. + 0.12 [–0.11 to + 0.54] mg/dL, *p* < 0.001), and the platelet count increase was higher in survivors (+ 35.0 [–20.8 to + 80.0] vs. + 8.5 [–35.0 to + 53.0] × 10^9^/L, *p* = 0.013). Also, we found the variation of bilirubin was significantly higher in non-survivors (+ 0.04 [–0.16 to + 0.4] vs. + 0.05 [–0.16 to + 0.56] mg/dL, *p* = 0.023). All these variations are detailed in Fig. 2 and Additional file 1: Table 5. There were no correlations between ventilatory ratio and prognostic biomarkers, lung mechanics or gas exchange data except weak, yet significant correlations were found between ventilatory ratio and PEEP (*r* = 0.11, *p* < 0.001), PaO_2_/FiO_2_(*r* =  − 0.19, *p* < 0.001) and driving pressure (*r* = 0.15, *p* = 0.004) at day 3 (Additional file 1: Fig. 3 and Table 6).

### Treatments and complications during ICU admission

Among the 1,118 patients, 986 (88.8%) patients received Hydroxychloroquine and 892 (80.4%) Lopinavir/Ritonavir during their ICU admission. Dexamethasone and methylprednisolone were used in 233 (21.4%) and 572 (52.4%) patients, respectively, while 499 (45.0%) were treated with Tocilizumab. Therapeutic anticoagulation with heparin was administered in 1065 (96.0%) patients. Those that survived to hospital discharge received this treatment more frequently [662 (97.1% of survivors) vs. 403 (94.4% of non-survivors); *p* = 0.04]. Coagulation disorders were present in 290 (26.0%) of the population, and hemorrhage complications were identified in 103 (9.2%) patients. Vasopressors were needed at any time of ICU admission in 1028 (92.6%) patients. Acute renal failure was identified in 493 (44.1%) patients, and 157 (14.1%) patients needed renal replacement therapy. Neuromuscular blockers were used at some point in 950 (85.9%) patients, and 482 (43.2%) patients needed tracheostomy. Recruitment maneuvers were used in 653 (61.8%) patients, prone positioning in 874 (79.0%), and in 20 (1.8%) cases extracorporeal respiratory support was needed. According to the Berlin definition, 76 (6.8%) had mild ARDS, 467 (41.8%) had moderate ARDS, and 575 (51.4%) had severe ARDS.

Although the vast majority of the cohort received antibiotic treatment (1102 patients, 99.2%), bacterial pneumonia was diagnosed in 368 (33.1%) of ventilated patients and bacteremia in 446 (40.0%). Thirty-three percent of patients had hepatic dysfunction. Pneumothorax [53(7.7%) vs 55 (12.7%), *p* = 0.007)] and hemorrhages [49(7.2%) vs 54 (12.7%), *p* = 0.004)] were more frequent in non-survivor patients. Additional file 1: Table 7 lists COVID-19 therapies and other treatments, and Additional file 1: Table 8 displays the major complications in this subpopulation of 1,118 patients.

### Outcomes and predictors of in-hospital mortality

The in-hospital mortality of all the COVID-19 patients admitted to an ICU during the study period was 32.6%, and that of patients needing intubation during the first 24 h was 40.2%.

In particular, in the cohort of patients still needing MV at day 3, we found an overall in-hospital mortality of 38.7%. Specifically on this subpopulation, ICU mortality was 37.5%, while 28-day mortality was 31.9%. The main causes of death were multiorgan failure (*n* = 179; 41.7%) and respiratory failure (*n* = 178; 41.5%). The duration of MV, ICU, and hospital stay was 16.0 [9.0–27.0], 20.0 [11.0–32.0], and 30.0 [19.0–48.0] days, respectively (Additional file 1: Table 9).

After multivariable adjustment including variables at day 1 of ICU admission, older age, the presence of chronic respiratory disease, higher levels of creatinine, and a higher ventilatory ratio were significantly associated with an increased risk for in-hospital mortality (Table 3). Equivalent results were obtained when died or extubated patients during these first three days were included in the analysis (Additional file 1: Table 10).

Likewise, when we included in the model the change in variables between day 1 and 3 of ICU admission, we found that a higher increase in the ventilatory ratio (OR 1.04 [CI 1.01–1.07]) and the creatinine levels (OR 1.05 [CI 1.01–1.09]) and a lower increase in the platelet count (OR 0.96 [CI 0.93–1.00]) were significantly associated with a higher risk of death. (Table 4). Interestingly, we found that neither the initial PaO_2_/FiO_2_ ratio (0.97 [CI 0.94 to 1.01]) nor its variation (OR 0.99 [CI 0.95 to 1.02]) were associated with in-hospital mortality.

## Discussion

The current study focused on a very homogeneous population of COVID-19 patients that needed intubation and mechanical ventilation at day 1 of ICU admission and continued to be mechanically ventilated at day 3. The in-hospital mortality of these patients in the first wave of the pandemic was 38.7%. We investigated the factors associated with mortality at day 1 and more importantly, any changes between day 1 and day 3. The day 1 model found that age, chronic respiratory disease, increased serum creatinine, and increased ventilatory ratio were independent factors associated with in-hospital mortality while the day 1 vs. day 3 model identified age, higher increase in serum creatinine from day 1 to day 3, lower increase in the number of platelets from day 1 to day 3, and higher increase in ventilatory ratio from day 1 to day 3 as predictors of mortality.

The reported percentage mortality of COVID patients admitted to the ICU is very heterogeneous, ranging from 30 to 60%, and mainly due to mixed noninvasively and invasively mechanically ventilated populations, mixed periods of the pandemic and different endpoints. Our multicenter study shows the in-hospital mortality of a very well-defined population that needed MV and remained ventilated 3 days after starting ventilation (38.7%).

At day 1, we found some demographic variables associated with higher mortality such as age and chronic respiratory diseases. These factors cannot be modified and simply reflect a more vulnerable population in whom COVID-19 has a worse evolution. Other studies confirm that age and chronic respiratory diseases are poor prognostic factors both in ventilated, mixed ventilated and non-ventilated patients [15, 16]. Among laboratory tests, only higher serum creatinine at ICU admission was associated with in-hospital mortality. Remarkably, oxygenation at day 1 of MV was not a factor associated with higher mortality. The most comparable study to our investigation is that of Botta et al., who studied 533 mechanically ventilated Dutch COVID patients [17]. Like us, they found that age was associated with 28-day mortality, while PaO_2_/FiO_2_ was not. In our study, the ventilatory ratio at day 1 was associated with in-hospital mortality. Botta et al. [17] did not measure this surrogate marker of dead space. However, they showed that higher tidal volume and lower compliance were associated with increased 28-day mortality.

In this context, determining prognostic factors is crucial to detect those that are amenable to medical intervention. In many acute respiratory diseases that need MV, day 3 of evolution is crucial. In both severe community-acquired pneumonia [18] and ventilator-associated pneumonia [5], the differences between day 1 and day 3 in key physiological parameters are reliable predictors of evolution and prognosis. Therefore, we performed a second multivariable model comparing day 1 and day 3 of MV. Overall, we found that the deltas (difference day 3 vs. day 1) in platelet counts (lower increase), creatinine levels (higher increase) and ventilatory ratio (higher increase) were significantly associated with in-hospital mortality. Changes in oxygenation were not associated with lower mortality.

In particular, the changes found in serum creatinine levels and platelets counts reflect organ failure in severe COVID patients. Since COVID-19 is a systemic disease that can affect all organs, renal failure can occur due to direct kidney injury [19]. Alterations in coagulation may have a significant impact on the platelet count, which is well described in severe COVID patients [19, 20]. Outstandingly, we found that increases in ventilatory ratio were associated with in-hospital mortality, while there was no evidence that changes in the degree of hypoxemia worsened the prognosis in our population of COVID-19 pneumonia ARDS patients.

The ventilatory ratio is a recently validated index that is appealing because it is simple to calculate using the minute ventilation and the PaCO_2_. It compares these two parameters to corresponding ideal and predicted values as a stand-in for Vd/Vt. This index has been validated in controlled modes of MV. The ventilatory ratio depends on CO_2_ production, and any increase in CO_2_ production can modify its value. A value approximating 1 would represent normal ventilating lungs [21]. In non-COVID ARDS patients, a high ventilatory ratio is associated with mortality [22, 23].

We have previously reported that the ventilatory ratio is elevated in COVID ARDS patients [24]. In a series of 267 COVID-ARDS patients, Schenck et al. [25] also found higher values in those patients that remained intubated at day 3 and 7. They also found significantly higher values in the 47 deceased patients (median ventilatory ratio of 2.26 [1.53–2.50]). In a sample of only 8 patients, Liu et al. [26] found the ventilatory ratio was lower in those patients that received lower tidal volumes. None of these three studies performed multivariable analyses of mortality. Finally, the Provent COVID data [27] including 927 consecutive mechanically ventilated patients shows that the quantification of the impairment of ventilation using dead space estimates (dead space fraction, ventilatory ratio, and end-tidal to arterial PCO_2_ ratio), neither at baseline nor in the following days, is not significantly associated with 28 day mortality. The exclusion or not of patients receiving ECMO was not specifically reported [27]. In addition, other important differences between this study and ours are that severity of ARDS was higher in our study ( 51% vs 10%) and that we have included delta differences between day 3 and day 1 which not seems to be the case in the Morales Quintero study [27].

Our multivariable regression models showed that an increase in ventilatory ratio at ICU admission was associated with in-hospital mortality, with each unit increasing risk-of-death by 7% ([1–14], *p* = 0.015). Delta of ventilatory ratio at day 3 was also associated with mortality, with each unit-increase rising risk-of-death by 5% ([1–10], *p* = 0.030). In comparison with previous studies in non-COVID ARDS patients, Sinha et al. found that ventilatory ratio was independently associated with in-hospital mortality after adjusting for PaO_2_/FiO_2_ ratio and driving pressure (OR 1.51 [CI 1.09–2.12), *p* = 0.015) [22]. It can be argued that our findings on ventilatory ratio could be expected after Sinha´s et al. results on non-COVID ARDS [22]. However, we think that they were necessary after all the controversies about ARDS COVID phenotypes. Another important difference compared to Sinha et al. [22] is that we demonstrated not only the prognostic value of ventilatory ratio at day 1 but the prognostic value at day 3 as well, which gives a lot consistency for our data.

Interpretation of the ventilatory ratio is complex, but it can be considered as a surrogate marker of physiologic dead space [22]. Increased dead space in COVID 19 may be due to a combination of hypoperfused alveoli due to microthrombosis of capillary alveoli plus interstitial edema that impairs pulmonary circulation [28]. In relation to the first mechanism (microthrombosis), neither we nor Barbeta et al. [24] found a correlation between the blood levels of D-dimer and the ventilatory ratio. In addition, we did not find significant correlations of VR with platelets, or compliance. We found a weak correlation with PaO_2_/FiO_2_. Sinha et al. also found a negative correlation between PaO_2_/FiO_2_ and ventilatory ratio, suggesting that ventilation–perfusion mismatch conduct to hypercarbia and hypoxemia [22]. Nevertheless, in our study ventilatory ratio exceeds PaO_2_/FiO_2_ in predicting in-hospital mortality. In fact, for its association to the extent of poorly aerated lung tissue [29], the physiologic dead space has been suggested as a stronger predictor than oxygenation of the non-COVID ARDS outcomes [30]. Unlike typical ARDS, the profound hypoxemia observed in COVID-19 might be found in patients with relatively preserved lung volumes and respiratory compliances [1], thus probably not reflecting the burden of parenchymal disease [31, 32].

Moreover, patients with higher dead space usually need higher minute ventilation to avoid hypercarbia, leading to an increase in lung injury due to higher dynamic mechanical power [33]. Indeed, the potential role of lung distension and ventilator-induced lung injury cannot be negligibly as the weak correlation between driving pressure and ventilatory ratio at day 3 might show. It is possible that an increasing ventilatory ratio is a consequence of the progression of disease, but on the other hand it could also be a parameter that we could measure to monitor the evolution of the patient when applying specific treatments and ventilatory strategies.

There are several limitations of this study. First, our measurements on day 1 and 3 are only snapshots of the dynamic nature of COVID 19 respiratory failure (we chose the worst value). Second, we did not measure volumetric capnography, and consequently, we were unable to assess the effects of metabolic rate on gas exchange. Metabolic rate can vary during fever and neuromuscular blockade. Third, and due to the observational nature of the study, the ventilation protocols were not standardized, but the majority of centers used protective ventilation strategies. Fourth, it has been shown that the ventilatory ratio is increased when HME filters are used in comparison with the use of heated humidifiers. HME filters were used in most of our patients. Finally, the lack of a control group of ARDS non-COVID-19 patients it is another potential limitation. However, to collect contemporary solid data of this population was totally impossible due to the very low number of non-COVID patients admitted during the study period in the different ICU's which could have made impossible a solid matching with COVID-19 ARDS patients.

The strengths of this study are its multicenter nature, the inclusion of a reasonably large number of patients with very homogeneous characteristics, and the performance of multivariable analysis on day 1 of MV and at day 3, allowing us to consider the magnitude of changes that took place between day 1 and 3.

## Conclusions

In conclusion, in a population of COVID 19 ARDS patients intubated at day 1 of ICU admission that remained ventilated after 3 days we found factors independently associated with in-hospital mortality both at day 1 and most importantly, changes that occurred between day 1 and day 3 that were predictors of outcome. Higher levels of creatinine and ventilatory ratio at day 1 and higher increase in ventilatory ratio and creatinine levels and lower increase in platelets at day 3 were independently associated with in-hospital mortality. We found that age and chronic respiratory diseases were also independently associated with in-hospital mortality. The PaO_2_/FiO_2_ ratio at ICU admission and its worsening or improvement at day 3 was not associated with prognosis. Further interventional and prospective studies are needed to determine treatments and strategies that could decrease the ventilatory ratio.

## Supplementary Information

**Additional file 1**. Supplementary tables and figures.

## Data Availability

The datasets used and/or analyzed during the current study are available from the corresponding author on reasonable request.

## Abbreviations

ARDS: Acute Respiratory Distress Syndrome
AUC: Area under the receiver operating characteristic curve
CI: Confidence interval
COVID-19: Coronavirus disease 2019
CRP: C-reactive protein
Crs: Compliance of the respiratory system
ECMO: Extracorporeal membrane oxygenation
FiO_2_: Fraction of inspired oxygen
ICU: Intensive care unit
MV: Invasive mechanical ventilation
OR: Odds ratio
PaCO_2_: Partial pressure of carbon dioxide in arterial blood
PaO_2_: Partial pressure of oxygen in arterial blood
PBW: Predicted body weight
PEEP: Positive end-expiratory pressure
SD: Standard deviation
SOFA: Sequential Organ Assessment Failure Score
STROBE: Strengthening the Reporting of Observational Studies in Epidemiology
VIF: Variance inflation factor
VILI: Ventilation-induced lung injury
VR: Ventilatory ratio
