# Supplementary material for: The evolution of the ventilatory ratio is a prognostic factor in mechanically ventilated COVID-19 ARDS patients
Source: Crit Care. 2021 Sep 13;25:331. doi: 10.1186/s13054-021-03727-x (PMC8436582; doi:10.1186/s13054-021-03727-x)
Supplement: Supplementary file 1 — Additional file 1. Supplementary tables and figures. [file 13054_2021_3727_MOESM1_ESM.docx]

**ADDITIONAL FILE 1**

**ONLINE SUPPLEMENTARY DATA**

**The EVOLUTION OF THE VENTILATORY RATIO IS A PROGNOSTIC FACTOR IN MECHANICALLY VENTILATED COVID-19 ARDS PATIENTS**

Antoni Torres, et al.

**FIGURE LEGEND**

**e-Figure 1.** Missing data map of variables including in the multivariant analyses. Missing observations are displayed in white while observations with valid values are shown in golden. The assessed variables are on the x-axis and the observations are on the y-axis.

**e-Figure 2.** Smoothed scatter plots between each continuous predictor variables and outcomes in logit scale.

**e-Figure 3.** Heatmap of pairwise correlation between biomarkers, lung mechanics, and gas exchange at ICU admission (A) and at day 3 (B). The color key of the Pearson’s coefficient correlations is shown on the right. Statistically significant correlations are indicated with green surrounding squares (p-value≤0.05).

**e-Table 1.** List of participating centers

| Site and Region |
| --- |
| Hospital Virgen del Rocío, Andalucía |
| Hospital Virgen De Valme, Andalucía |
| Hospital San Juan de Dios, Andalucía |
| Hospital Virgen Macarena, Andalucía |
| Hospital Universitario de Jerez de la Frontera, Andalucía |
| Hospital Universitario Reina Sofía, Andalucía |
| Hospital Nuestra Señora de Gracia, Aragón |
| Hospital Universitario de Gran Canaria Doctor Negrín, Canarias |
| Hospital Universitario Marqués de Valdecilla, Cantabria |
| Hospital Universitario Río Hortega, Castilla y León |
| Hospital Clínico Universitario de Valladolid, Castilla y León |
| Hospital General Río Carrión, Castilla y León |
| Hospital Germans Trias i Pujol, Catalunya |
| Hospital Universitario Vall d'Hebron, Catalunya |
| Hospital Clinici Provinvial Barcelona, Catalunya |
| Hospital Universitari Bellvitge, Catalunya |
| Hospital Universitario del Mar, Catalunya |
| Hospital Universitari Mútua Terrassa, Catalunya |
| Hospital Universitari Arnau de Vilanova, Catalunya |
| Hospital Universitari Joan XXIII de Tarragona, Catalunya |
| Hospital Sagrat Cor, Catalunya |
| Hospital Parc Taulí, Catalunya |
| Clínica Sagrada Familia, Catalunya |
| Hospital de Tortosa Verge de la Cinta, Catalunya |
| Hospital de Mataró, Catalunya |
| Hospital de Santa Maria, Catalunya |
| Hospital General Universitario Gregorio Marañón, Comunidad de Madrid |
| HM Hospitales Madrid, Comunidad de Madrid |
| Hospital Universitario La Paz, Comunidad de Madrid |
| Hospital Universitario Ramón y Cajal, Comunidad de Madrid |
| Hospital Universitario de La Princesa, Comunidad de Madrid |
| Hospital Infanta Leonor de Madrid, Comunidad de Madrid |
| Hospital Universitario 12 de Octubre, Madrid |
| Hospital Universitario Sant Joan d’Alacant, Comunitat Valenciana |
| Hospital Universitario La Fe de Valencia, Comunitat Valenciana |
| Hospital Clínic Universitari de València, Comunitat Valenciana |
| Hospital Universitario de Cruces, Euskadi |
| Hospital San Pedro de Alcántara, Extremadura |
| Hospital Álvaro Cunqueiro, Galicia |
| Hospital Universitario Lucus Augusti, Galicia |
| Hospital Clínico Universitario de Santiago, Galicia |
| Hospital Universitari Son Espases, Illes Balears |
| Hospital Universitari Son Llàtzer, Illes Balears |
| Hospital Universitario Central de Asturias, Principado de Asturias |

**e-Table 2.** Box- tidwell test for checking the linearity assumption of the positive variables. Samples with negative or zero value were replaced by 0.01.

| Variables | p-value |
| --- | --- |
| (Intercept) | **0.75** |
| PaO2/FiO2 | 0.71 |
| Log (PaO2/FiO2) | **0.99** |
| SOFA_hemo | 0.72 |
| Log (SOFA_hemo) | **0.63** |
| D-Dimer | 0.47 |
| Log (D-Dimer) | **0.034** |
| Platelets | 0.18 |
| Log (Platelets) | **0.17** |
| Creatinine | 0.23 |
| Log (Creatinine) | **0.54** |
| Age | 0.87 |
| Log (Age) | **0.99** |
| Lymphocytes | 0.45 |
| Log (Lymphocytes) | **0.032** |
| Ventilatory Ratio | 0.12 |
| Log (Ventilatory Ratio) | **0.12** |
| Total Bilirubin | 0.77 |
| Log (Total Bilirubin) | **0.81** |
| PaO2/FiO2:log (PaO2/FiO2) | 0.67 |
| SOFA_hemo:log (SOFA_hemo) | **0.75** |
| D-Dimer:log(D-Dimer) | 0.52 |
| Platelets:log (Platelets) | **0.18** |
| Creatinine:log (Creatinine) | 0.23 |
| Age:log (Age) | **0.84** |
| Lymphocytes:log (Lymphocytes) | 0.63 |
| Ventilatory Ratio:log (Ventilatory Ratio) | **0.13** |
| Total Bilirubin:log (Total Bilirubin) | 0.86 |

**e-Table 3:** Number of patients per center included in this subpopulation of 1,118 patients (COVID 19 ARDS patients intubated at day 1 of ICU admission that remained ventilated after 3 days).

| Included patients | Sites  (N=44) |
| --- | --- |
| 1 – 25 | 27 (61%) |
| 25 – 50 | 6 (14%) |
| 51 – 75 | 9 (21%) |
| 76 – 100 | 1 (2%) |
| > 100 | 1 (2%) |

**e-Table 4.** Demographic and clinical characteristics at ICU admission of patients that received invasive mechanical ventilation (MV) at any time of ICU stay.

|  | **No.** | **All patients (n=1645)** | **Survivors**  **(n=1016)** | **Non-survivors**  **(n=629)** | **p-value** |
| --- | --- | --- | --- | --- | --- |
| **Age, years** | 1645 | 64.0 [56.0-71.0] | 61.0 [53.0-68.0] | 68.0 [61.0-73.0] | **<0.001** |
| **Age, categories** | 1645 |  |  |  |  |
| <50 |  | 236 (14.4%) | 199 (19.6%) | 37 (5.9%) | **<0.001** |
| 50–69 |  | 910 (55.3%) | 593 (58.4%) | 317 (50.4%) | **0.002** |
| >70 |  | 434 (26.38%) | 191 (18.8%) | 243 (38.6%) | **<0.001** |
| **Sex, female** |  | 471 (28.63%) | 315/1016 (31.0%) | 156/629 (24.8%) | **0.007** |
| **BMI, kg/m**^2^ | 1487 | 28.3 [25.7-31.8] | 28.6 [25.7-32.0] | 28.0 [25.6-31.3] | **0.1** |
| Comorbidities |  |  |  |  |  |
| Active smoker | 1069 | 84 (7.9%) | 44 (6.5%) | 40 (10.2%) | **0.034** |
| Hypertension | 1644 | 820 (49.9%) | 457 (45.0%) | 363 (57.8%) | **<0.001** |
| Diabetes mellitus | 1644 | 375 (22.8%) | 209 (20.6%) | 166 (26.4%) | **0.006** |
| Dyslipidemia | 1644 | 375 (22.8%) | 223 (22.0%) | 152 (24.2%) | 0.30 |
| Chronic cardiac failure | 1643 | 195 (11.9%) | 91 (9.0%) | 104 (16.6%) | **<0.001** |
| Chronic kidney disease | 1644 | 94 (5.7%) | 40 (3.9%) | 54 (8.6%) | **<0.001** |
| Chronic respiratory disease | 1643 | 170 (10.4%) | 78 (7.7%) | 92 (14.7%) | **<0.001** |
| **Days since first symptoms** | 1611 | 7.0 [5.0-9.0] | 7.0 [5.0-9.0] | 7.0 [4.0-9.0] | **0.008** |
| Days from hospital admission to ICU admission | 1633 | 2.0 [0.0-4.0] | 2.0 [0.0-4.0] | 2.0 [0.0-5.0] | 0.56 |
| APACHE score | 907 | 12.0 [9.0-15.0] | 11.0 [8.0-14.5] | 13.0 [11.0-17.0] | **<0.001** |
| SOFA score | 1102 | 6.0 [4.0-8.0] | 6.0 [3.0-8.0] | 7.0 [4.0-8.0] | **<0.001** |
| SOFA hemodynamic component | 1516 | 3.0 [0.0-4.0] | 1.0 [0.0-4.0] | 3.0 [0.0-4.0] | **0.013** |
| SOFA renal component | 1619 | 0.0 [0.0-0.0] | 0.0 [0.0-0.0] | 0.0 [0.0-1.0] | **<0.001** |
| Temperature, ºC | 1462 | 37.0 [36.1-37.9] | 37.0 [36.2-37.9] | 36.9 [36.0-37.7] | **0.01** |
| Respiratory rate, bpm | 1376 | 25.0 [20.0-30.3 | 25.0 [20.0-31.3] | 25.0 [20.0-30.0] | 0.73 |
| Respiratory support |  |  |  |  |  |
| High-flow oxygen | 1616 | 616 (38.1%) | 414 (41.3%) | 202 (32.9%) | **0.001** |
| Noninvasive MV | 1629 | 177/1629 (10.9%) | 99 (9.8%) | 78 (12.5%) | 0.10 |
| Invasive MV | 1637 | 1227 (75.0%) | 739 (73.2%) | 488 (78.1%) | **0.026** |
| Arterial blood gases | | | | | |
| PaO_2_/FIO_2_ ratio, mmHg | 1480 | 112.6 [78.2-168.1] | 115.7 [81.4-174.0] | 105.2 [72.3-156.4] | **0.001** |
| PaO_2_/F_I_O_2_ ratio categories | 1480 |  |  |  |  |
| PaO_2_/FIO_2_ ratio <= 100 |  | 610 (41.2%) | 355 (38.2%) | 255 (46.4%) | **0.002** |
| PaO_2_/FIO_2_ ratio 101-200 |  | 624 (42.2%) | 408 (43.9%) | 216 (39.3%) | 0.09 |
| PaO_2_/FIO_2_ ratio 201-300 |  | 172 (11.6%) | 113 (12.2%) | 59 (10.7%) | 0.45 |
| PaO_2_/FIO_2_ ratio > 301 |  | 62 (4.2%) | 45 (4.8%) | 17 (3.1%) | 0.11 |
| pH | 1551 | 7.39 [7.31-7.45] | 7.4 [7.33-7.45] | 7.36 [7.29-7.43] | **<0.001** |
| PaCO_2_, mmHg | 1541 | 41.4 [35.0-50.0] | 40.0 [34.6-48.0] | 43.0 [35.5-53.9] | **<0.001** |
| Lactate, mg/dL | 1081 | 12.9 [9.9-17.6] | 12.6 [9.4-16.2] | 14.4 [10.8-19.8] | **<0.001** |
| Laboratory findings | | | | | |
| Lymphocyte count, 10^9/L | 1593 | 0.68 [0.46-0.94] | 0.7 [0.5-1.0] | 0.61 [0.4-0.9] | **<0.001** |
| Platelet count, 10^9/L | 1613 | 224.0 [169.0-294.0] | 234.0 [174.0-299.0] | 212.0 [160.0-281.3] | **<0.001** |
| D-dimers, mg/L | 1253 | 1.07 [0.57-3.17] | 0.96 [0.52-2.19] | 1.47 [0.67-5.37] | **<0.001** |
| Ferritin, ng/mL | 572 | 1359 [781-2260] | 1266 [689-2142] | 1465 [846-2376] | 0.07 |
| IL6, pg/mL | 360 | 101.6 [46.3-177.2] | 93.8 [44.9-173.0] | 124.0 [59.4-193.3] | 0.15 |
| CRP, mg/dL | 1457 | 16.9 [9.2-26.1] | 16.4 [9.1-25.5] | 17.7 [9.3-27.2] | 0.09 |
| Bilirubin, mg/dL | 1454 | 0.63 [0.42-1.0] | 0.64 [0.43-1.0] | 0.6 [0.4-1.0] | 0.63 |
| Serum creatinine, mg/dL | 1619 | 0.87 [0.68-1.15] | 0.82 [0.65-1.04] | 0.97 [0.74-1.31] | **<0.001** |

e-Table 4 caption: Continuous variables are expressed as median (IQR) and categorical variables as number (percentages). CRP, C-reactive protein; FiO_2_, fraction of inspired oxygen; MV, mechanical ventilation; PaCO_2_, arterial partial pressure of carbon dioxide; PaO_2_, partial pressure of arterial oxygen; SOFA: sequential organ failure assessment score.

**e-Table 5.** Delta differences between day 3 and ICU admission of laboratory findings and ventilation management variables of the early ventilated patients that remained ventilated after 2 days according to in-hospital mortality.

|  | **No.** | **All patients (n=1118)** | **Survivors**  **(n=685)** | **Non-survivors**  **(n=433)** | **p-value** |
| --- | --- | --- | --- | --- | --- |
| Arterial blood gases | | | | | |
| PaO_2_/FIO_2_ ratio, mmHg | 1006 | +46.7 [-9.2-108.3] | +52.5 [-3.9-114.4] | +38.4 [-15.3-96.7] | 0.07 |
| pH | 1056 | +0.02 [-0.05-0.10] | +0.03 [-0.04-0.10] | +0.02 [-0.06-0.11] | 0.15 |
| PaCO_2_, mmHg | 1068 | +3.1 [-5.1-12.0] | +2.3 [-5.4-10.0] | +4.3 [-5.0-13.1] | **0.007** |
| Lactate, mg/dL | 650 | +1.8 [-2.2-6.8] | +1.8 [-1.8-7.1] | +.7 [-3.0-6.3] | 0.93 |
| Laboratory findings | | | | | |
| Lymphocyte count, 10^9/L | 1081 | 0.00 [-0.20-0.26] | 0.00 [-0.20-0.30] | 0.00 [-0.20-0.20] | **0.041** |
| Neutrophil count, 10^9/L | 472 | 0.00 [-3.3-2.37] | -0.02 [-3.05-2.27] | +0.02 [-3.58-2.65] | 0.95 |
| Platelet count, 10^9/L | 1088 | +24.0 [-30.0-75.0] | +35.0 [-20.8-80.0] | +8.5 [-35.0-53.0] | **0.013** |
| D-dimers, mg/L | 660 | +0.45 [-0.18-2.08] | +0.40 [-0.11-1.69] | +0.58 [-0.36-3.67] | **0.001** |
| Ferritin, ng/mL | 217 | -51.0 [-368.0-303.0] | -38.5 [-336.8-273.5] | -79.0 [-370.0-314.5] | 0.66 |
| IL6, pg/mL | 65 | -0.6 [-50.3-75.9] | -2.3 [-51.3-42.7] | +2.0 [-22.8-105.4] | 0.93 |
| CRP, mg/dL | 859 | -4.6 [-14.04-3.4] | -4.8 [-14.8-3.7] | -4.16 [-13.2-2.5] | 1.00 |
| Bilirubin, mg/dL | 906 | +0.04 [-0.16-0.43] | +0.04 [-0.16-0.40] | +0.05 [-0.16-0.56] | **0.023** |
| Serum creatinine, mg/dL | 1094 | +0.02 [-0.13-0.32] | 0.0 [-0.14-0.22] | +0.12 [-0.11-0.54] | **<0.001** |
| Ventilatory setting and pulmonary mechanics | | | | | |
| Tidal volume/PBW (ml/kg) | 445 | 0.0 [-0.52-0.63] | +0.02 [-0.36-0.67] | 0.0 [-0.67-0.54] | 0.74 |
| Respiratory rate, rpm | 945 | 0.0 [-2.0-2.0] | 0.0 [-2.0-2.0] | 0.0 [-1.0-3.0] | 0.09 |
| PEEP, cmH_2_O | 1014 | 0.0 [-2.0-1.0] | 0.0 [-2.0-1.0] | 0.0 [-2.0-2.0] | 0.23 |
| FiO_2_, % | 1036 | -20.0 [-35.0--5.0] | -20.0 [-40.0--10.0] | -17.0 [-30.0-0.0] | 0.11 |
| Peak inspiratory pressure, cmH_2_O | 453 | 0.0 [-3.0-3.0] | 0.0 [-3.9-3.0] | 0.0 [-3.0-4.0] | 0.68 |
| End-inspiratory plateau pressure, cmH_2_O | 310 | -0.7 [-3.0-2.0] | -1.0 [-3.0-1.0] | 0.0 [-3.0-2.1] | 0.25 |
| Driving pressure, cmH_2_O^a^ | 296 | 0.0 [-3.0-2.0] | 0.0 [-3.0-2.0] | 0.0 [-2.0-3.0] | 0.39 |
| Compliance, mL/cmH_2_O^b^ | 274 | +0.69 [-6.28-8.79] | +1.3 [-6.28-9.39] | +0.11 [-6.19-7.17] | 0.18 |
| Ventilatory ratio^c^ | 715 | +0.18 [-0.18-0.56] | +0.14 [-0.18-0.47] | +0.32 [-0.17-0.75] | **<0.001** |

e-Table 5 caption: Delta was calculated as variable at day 3 minus variable at ICU admission. Continuous variables are expressed as median (IQR). CRP, C-reactive protein; FiO_2_, fraction of inspired oxygen; PaCO_2_, arterial partial pressure of carbon dioxide; PaO_2_, partial pressure of arterial oxygen; PBW, predicted body weight. ^a^ Defined as plateau pressure—PEEP. ^b^ Defined as tidal volume/(Plateau pressure − PEEP). ^c^ Defined as (minute ventilation × PaCO_2_)/(PBW × 100 × 37.5)

**e-Table 6.** Correlations between ventilatory ratio and main biomarkers, lung mechanics or gas exchange data at ICU admission and day 3.

| Variable 1 | Variable 2 | Correlation coefficient | p-value |
| --- | --- | --- | --- |
| Ventilatory ratio at ICU admission | PaO_2_/ FiO_2_ at ICU adm. | 0.01 | 0.68 |
|  | Platelets at ICU adm. | -0.02 | 0.61 |
|  | D-Dimer at ICU adm. | -0.004 | 0.90 |
|  | PEEP at ICU adm. | -0.03 | 0.43 |
|  | Driving pressure at ICU adm. | 0.02 | 0.68 |
|  | Compliance at ICU adm. | -0.02 | 0.66 |
| Ventilatory ratio at day 3 | PaO_2_/ FiO_2_ at day 3 | **-0.19** | **<0.001** |
|  | Platelets at day 3 | -0.06 | 0.07 |
|  | D-Dimer at day 3 | 0.02 | 0.6 |
|  | PEEP at day 3 | **0.11** | **<0.001** |
|  | Driving pressure at day 3 | **0.14** | **0.004** |
|  | Compliance at day 3. | -0.05 | 0.324 |

**e-Table 7.** Main interventions and treatments in the subpopulation of 1,118 patients (COVID-19 ARDS patients intubated at day 1 of ICU admission that remained ventilated after 3 days) according to in-hospital mortality.

|  | | **No.** | **All patients (n=1118)** | | **Survivors**  **(n=685)** | | **Non-survivors**  **(n=433)** | | **p-value** |
| --- | --- | --- | --- | --- | --- | --- | --- | --- | --- |
| COVID-19 therapies | 1110 | | |  | |  | |  | |
| Ribavirin | |  | 2 (0.2%) | | 1 (0.2%) | | 1 (0.2%) | | 1 |
| Lopinavir/ritonavir | |  | 892 (80.4%) | | 555 (81.3%) | | 337 (78.9%) | | 0.35 |
| Remdesivir | |  | 84 (7.6%) | | 54 (7.9%) | | 30 (7.0%) | | 0.67 |
| Interferon alpha | |  | 9 (0.8%) | | 1 (0.2%) | | 8 (1.9%) | | **0.003** |
| Interferon beta | |  | 488 (44.0%) | | 289 (42.3%) | | 199 (46.6%) | | 0.17 |
| Chloroquine | |  | 63 (5.7%) | | 38 (5.6%) | | 25 (5.8%) | | 0.89 |
| Hydroxychloroquine | |  | 986 (88.8%) | | 612 (89.3%) | | 373 (86.1%) | | 0.24 |
| Tocilizumab | |  | 499 (45.0%) | | 324 (47.4%) | | 175 (41.0%) | | 0.05 |
| Darunavir/cobicistat | |  | 23 (2.1%) | | 13 (1.9%) | | 10 (2.3%) | | 0.67 |
| Pharmacological adjunctive  therapies | | | | | | | | | |
| Continuous furosemide | | 1002 | 701 (63.6%) | | 412 (60.9%) | | 289 (68.0%) | | **0.017** |
| Inmmunoglobulins | | 1003 | 13 (1.2%) | | 7 (1.0%) | | 6 (1.4%) | | 0.58 |
| Subcutaneous heparin | | 1009 | 1065 (96.0%) | | 662 (97.1%) | | 403 (94.4%) | | **0.039** |
| ≤ 1 mg/kg/day | | 1009 | 816 (73.6%) | | 527 (77.3%) | | 289 (67.7%) | | **<0.001** |
| > 1 mg/kg/day | | 1009 | 381 (34.4%) | | 234 (34.3%) | | 147 (34.4%) | | 1.00 |
| Convalescent plasma | | 1105 | 6 (0.5%) | | 5 (0.7%) | | 1 (0.2%) | | 0.47 |
| Vasopressor treatment | | 1110 | 1028 (92.6%) | | 608 (89.3%) | | 420 (97.9%) | | **<0.001** |
| Continuous neuromuscular blockers | | | 950 (85.9%) | | 561 (82.5%) | | 389 (91.2%) | | **<0.001** |
| Corticosteroid | | 1092 | 831 (76.1%) | | 504 (74.6%) | | 327 (78.6%) | | 0.14 |
| Other adjunctive treatments | | | | | | | | | |
| Tracheostomy | | 1115 | 482 (43.2%) | | 326 (47.7%) | | 156 (36.1%) | | **<0.001** |
| Recruitment maneuvers | | 1057 | 653 (61.8%) | | 371 (57.3%) | | 282 (68.8%) | | **<0.001** |
| Prone position | | 1107 | 874 (79.0%) | | 509 (74.7%) | | 365 (85.7%) | | **<0.001** |
| Prone length, hours | | 812 | 48.0 [25.8-82.0] | | 48.0 [24.0-72.0] | | 60.0 [36.0-90.0] | | **<0.001** |
| ECMO support | | 1105 | 20 (1.8%) | | 10 (1.5%) | | 10 (2.4%) | | 0.36 |
| ECMO length, days | | 20 | 10.5 [4.8-16.5] | | 17.0 [11.0-24.5] | | 6.5 [4.0-10.3] | | **0.024** |
| Renal replacement therapy | | 1115 | 157 (14.1%) | | 62 (9.1%) | | 95 (22.0%) | | **<0.001** |

**e-Table 8.** Major complications in the subpopulation of 1,118 patients (COVID-19 ARDS patients intubated at day 1 of ICU admission that remained ventilated after 3 days) according to in-hospital mortality.

|  | **No.** | **All patients (n=1118)** | **Survivors**  **(n=685)** | **Non-survivors**  **(n=433)** | **p-value** |
| --- | --- | --- | --- | --- | --- |
| Bacterial pneumonia ^a^ | 1113 | 368 (33.1%) | 241 (35.2%) | 127 (29.6%) | 0.06 |
| ARDS ^b^ | 1118 |  |  |  |  |
| Mild |  | 76 (6.8%) | 65 (9.5%) | 11 (2.5%) | <0.001 |
| Moderate |  | 467 (41.8%) | 341 (49.8%) | 126 (29.1%) | <0.001 |
| Severe |  | 575 (51.4%) | 279 (40.7%) | 296 (68.4%) | <0.001 |
| Pneumothorax | 1118 | 108 (9.7%) | 53 (7.7%) | 55 (12.7%) | 0.007 |
| Pleural effusion | 1117 | 132 (11.8%) | 87 (12.7%) | 45 (10.4%) | 0.26 |
| Organizing pneumonia | 1092 | 54 (5.0%) | 38 (5.7%) | 16 (3.8%) | 0.20 |
| Tracheobronchitis | 1112 | 16 (1.4%) | 15 (2.2%) | 1 (0.2%) | 0.008 |
| Pulmonary embolism | 1083 | 94 (8.7%) | 71 (10.6%) | 23 (5.6%) | 0.005 |
| Myocardial infarction | 1117 | 132 (11.8%) | 87 (12.7%) | 45 (10.4%) | 0.26 |
| Endocarditis | 1118 | 9 (0.8%) | 4 (0.6%) | 5 (1.2%) | 0.32 |
| Myocarditis/pericarditis | 1116 | 3 (0.3%) | 1 (0.2%) | 2 (0.5%) | 0.56 |
| Cardiomyopathy | 1118 | 24 (2.2%) | 12 (1.8%) | 12 (2.8%) | 0.29 |
| Heart failure | 1117 | 27 (2.4%) | 19 (2.8%) | 8 (1.9%) | 0.43 |
| Cardiac ischemia | 1118 | 43 (3.9%) | 23 (3.4%) | 20 (4.6%) | 0.34 |
| Bacteremia | 1115 | 446 (40.0%) | 261 (38.2%) | 185 (42.9%) | 0.12 |
| Stroke | 1116 | 35 (3.1%) | 22 (3.2%) | 13 (3.0%) | 1.00 |
| Delirium | 1113 | 280 (25.2%) | 239 (34.9%) | 41 (9.6%) | <0.001 |
| Coagulation disorder ^c^ | 1116 | 290 (26.0%) | 158 (23.1%) | 132 (30.6%) | 0.006 |
| Disseminated intravascular coagulation ^d^ | 1102 | 69 (6.3%) | 34 (5.0%) | 35 (8.2%) | 0.041 |
| Anemia ^e^ | 1118 | 800 (71.6%) | 496 (72.4%) | 304 (70.2%) | 0.45 |
| Rhabdomyolysis | 1111 | 46 (4.1%) | 27 (4.0%) | 19 (4.4%) | 0.76 |
| Acute renal failure ^f^ | 1118 | 493 (44.1%) | 238 (34.7%) | 255 (58.9%) | <0.001 |
| Pancreatitis | 1118 | 14 (1.3%) | 8 (1.2%) | 6 (1.4%) | 0.79 |
| Liver dysfunction | 1114 | 369 (33.1%) | 218 (31.8%) | 151 (35.2%) | 0.27 |
| Hemorrhage | 1116 | 103 (9.2%) | 49 (7.2%) | 54 (12.5%) | 0.004 |

Definitions: ^a^ Clinically or radiologically diagnosed bacterial pneumonia managed with antimicrobials. Bacteriological confirmation was not required. ^b^ Acute Respiratory Distress Syndrome (ARDS) was defined according to Berlin criteria. Mild ARDS, PaO2/FiO2 >200 mmHg and ≤300 mmHg, with PEEP≥5 cm H_2_O.Moderate ARDS, PaO2/FiO2 >100 mmHg and ≤200 mmHg with PEEP ≥5 cm H_2_O. Severe ARDS, PaO2/FiO2 ≤100 mmHg with PEEP ≥5 cm H_2_O. ^c^ Abnormal coagulation was identified by abnormal prothrombin time or activated partial thromboplastin time. ^d^ Disseminated intravascular coagulation was defined by thrombocytopenia, prolonged prothrombin time, low fibrinogen, elevated D-dimer and thrombotic microangiopathy. ^e^ Hemoglobin consistently below 120 g/L for non-pregnant women and 130 g/L for men. ^f^ Acute renal injury was defined as an increase in serum creatinine by ≥0.3 mg/dL within 48 hours or an increase in serum creatinine to ≥1.5 times baseline.

**e-Table 9.** Main secondary outcomes in the subpopulation of 1,118 patients (COVID-19 ARDS patients intubated at day 1 of ICU admission that remained ventilated after 3 days) according to in-hospital mortality.

|  | **No.** | **All patients (n=1118)** | **Survivors**  **(n=685)** | **Non-survivors**  **(n=433)** | **p-value** |
| --- | --- | --- | --- | --- | --- |
| Length of ICU stay, days | 1116 | 20.0 [11.0-32.0] | 22.0 [13.0-35.0] | 16.0 [9.0-26.0] | <0.001 |
| Length of hospital stay, days | 1118 | 30.0 [19.0-48.0] | 38.0 [25.0-57.0] | 19.0 [11.0-30.0] | <0.001 |
| NIMV length, days | 263 | 2.0 [1.0-5.0] | 3.0 [1.0-6.0] | 2.0 [1.0-4.0] | 0.15 |
| IMV length, days | 1089 | 16.0 [10.0-27.0] | 16.0 [10.0-27.0] | 16.0 [9.0-25.0] | 0.16 |
| ICU mortality | 1118 | 419 (37.5) |  | 419 (97.0%) |  |
| Ventilator free days | 567 |  | 12.0 [0.0-18.0] |  |  |
| ICU free days | 1116 | 0.0 [0.0-10.0] | 6.0 [0.0-15.0] | 0.0 [0.0-0.0] | <0.001 |
| Cause of death | 429 |  |  |  |  |
| Respiratory failure |  |  |  | 178 (41.5%) |  |
| Septic shock |  |  |  | 22 (5.1%) |  |
| Multiorgan failure |  |  |  | 179 (41.7%) |  |
| Cardiovascular accident |  |  |  | 6 (1.4%) |  |
| 28-day mortality | 1118 | 357 (31.9%) |  | 357 (82.4%) | <0.001 |

e-Table 9 caption: ICU, intensive care unit; IMV, invasive mechanical ventilation; NIMV, non-invasive mechanical ventilation.

**e-Table 10. Multivariable model assessing predictors of in-hospital mortality (N=662 patients)**

| Variable | Odds ratio (95% CI) | p-value |
| --- | --- | --- |
| Age, years | 1.01 (1.01 to 1.02) | **<0.001** |
| Sex, male | 1.06 (0.98 to 1.14) | 0.16 |
| Hypertension | 1.00 (0.93 to 1.08) | 0.96 |
| Chronic respiratory disease | 1.09 (1.00 to 1.23) | **0.049** |
| SOFA hemodynamic component | 1.00 (0.98 to 1.02) | 0.82 |
| PaO_2_/F_I_O_2_ ratio at ICU admission, mmHg | 1.00 (1.00 to 1.00) | 0.12 |
| Serum creatinine at ICU admission, mg/dL | 1.09 (1.04 to 1.14) | **<0.001** |
| Lymphocyte count at ICU admission, x 10^9^/L | 1.00 (0.98 to 1.03) | 0.85 |
| Platelet count at ICU admission, x 10^9^/L | 1.00 (1.00 to 1.00) | 0.10 |
| Total bilirubin at ICU admission, mg/dL | 0.96 (0.91 to 1.02) | 0.22 |
| D-dimers at ICU admission, μg/L | 1.00 (1.00 to 1.00) | 0.23 |
| Ventilatory ratio at ICU admission | 1.08 (1.03 to 1.15) | **0.004** |

e-Table 10 caption: Mixed-effects model with centers as a random effect and considering a binomial distribution. AUC statistic (area under the curve) is 0.79 (95% CI 0.76 to 0.83) and Brier score is 0.18. CI, confidence interval; FiO_2_, fraction of inspired oxygen; ICU, intensive care unit; PaO_2_, partial pressure of arterial oxygen; SOFA, sequential organ failure assessment score.

**e-Figure 1**

e-Figure 1 caption. Missing data map of variables including in the multivariant analyses. Missing observations are displayed in white while observations with valid values are shown in golden. The assessed variables are on the x-axis and the observations are on the y-axis.

**e-Figure 2**

**
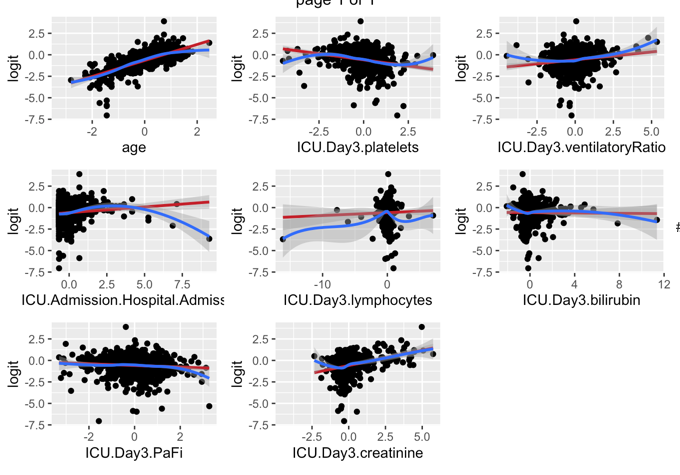
**

e-Figure 2 caption. Smoothed scatter plots between each continuous predictor variables and outcomes in logit scale.

**e-Figure 3**

e-Figure 3 caption. Heatmap of pairwise correlation between biomarkers, lung mechanics, and gas exchange at ICU admission (A) and at day 3 (B). The color key of the Pearson’s coefficient correlations is shown on the right. Statistically significant correlations are indicated with green surrounding squares (p-value≤0.05).
